# Composting Poultry Feathers with Keratinolytic *Bacillus subtilis*: Effects on Degradation Efficiency and Compost Maturity

**DOI:** 10.3390/ma18204667

**Published:** 2025-10-11

**Authors:** Justyna Sobolczyk-Bednarek, Anna Choińska-Pulit, Wojciech Łaba

**Affiliations:** 1 “Poltegor-Institute”, Opencast Mining Institute, Parkowa 25, 51-616 Wrocław, Poland; justyna.sobolczyk@igo.wroc.pl; 2 Department of Biotechnology and Food Microbiology, Faculty of Biotechnology and Food Science, Wrocław University of Environmental and Life Sciences, Chełmońskiego 37, 51-630 Wrocław, Poland; wojciech.laba@upwr.edu.pl

**Keywords:** solid state culture, keratinases, proteases, feathers, composting, compost maturity, *Bacillus subtilis*

## Abstract

The continuous advancement of the food industry is accompanied by increased generation of animal waste, including poultry feathers. Composting presents a sustainable alternative to disposal methods such as incineration by converting waste into valuable fertilizer products. This study aimed to evaluate the impact of inoculation with the keratinolytic strain *Bacillus subtilis* P22 on the quality and maturity of compost produced from feathers combined with organic additives (wood shavings and lignite). The experiment involved evaluation of the keratinolytic potential of the tested strain, and characterization of its proteolytic enzymes, solid-state cultures and composting conducted at semi-technical scale. The *B. subtilis* P22 strain demonstrated the ability to solubilize 78% of feather material within 7 days of cultivation. The keratinolytic enzyme complex was likely dominated by polycatalytic alkaline serine proteases, i.e., subtilisins. The effectiveness of the inoculum was confirmed in laboratory solid-state cultures, where the dry mass loss in inoculated samples was twice that of the control containing only endogenous microflora. At the semi-technical scale, inoculation with *B. subtilis* P22 significantly accelerated compost maturation and mineralization (C/N = 10.2; N-NH_4_^+^/N-NO_3_^−^ = 0.4; Cw/Corg = 0.9) compared to the control. The final compost’s mineral composition indicates its potential for use as an organic soil amendment.

## 1. Introduction

Each year, the poultry and meat industry generates millions of tons of organic waste, including feathers, bristles, hair, horns, and hooves. In recent decades, the poultry sector of the food industry has experienced substantial expansion, driven primarily by global population growth [1]. In Poland, poultry meat consumption has increased by 32% over the past five years [2]. This growth has led to a corresponding rise in slaughter by-products, such as feathers, which have garnered attention both for their potential valorization and because conventional disposal practices often pose significant environmental risks [3,4].

Feathers consist predominantly of keratin, a structural protein characterized by extensive cross-linking, hydrogen bonding, and disulfide bridges, which confer high resistance to biodegradation [5]. Conventional degradation techniques, such as acid or alkali hydrolysis, require the use of hazardous chemicals and pressure steam treatment, although effective, is energy-intensive and causes the degradation of heat-sensitive essential amino acids, i.e., lysine, methionine, and tryptophan, thereby diminishing the nutritional value of the product [3,6,7]. Furthermore, improper storage of feather waste can result in the release of volatile organic compounds, ammonia, hydrogen sulfide, and other malodorous gases, as well as leachates, which can contaminate surrounding environments and pose significant ecological risks [8].

An alternative approach to conventional feather degradation methods is the application of keratinolytic enzymes, which are produced by a variety of microorganisms, including bacteria, actinomycetes, and filamentous fungi [9,10,11,12,13]. Keratinase, a key enzyme in this group, is typically secreted extracellularly in response to keratin substrates and functions effectively across a wide range of pH and temperatures. It is most commonly classified as a serine protease, particularly within the subtilisin family (EC 3.4.21.x), although keratinolytic activity has also been observed among metalloproteases (EC 3.4.24.x) and cysteine proteases (EC 3.4.22.x), reflecting its biochemical diversity [3]. Most keratinases exhibit optimal activity at pH 6–9 and temperatures between 30 and 50 °C, although some thermostable variants function well beyond these ranges [14]. Keratinases play a crucial role in the bioconversion of keratinous waste into value-added products, with applications in bioremediation, agriculture, feed and food processing, pharmaceuticals, and the leather industry [15,16].

Understanding the specific types of keratinolytic enzymes and their optimal catalytic conditions is essential for enhancing the efficiency of keratin hydrolysis, which directly influences the economic viability and scalability of the process. Enzymatic catalysis offers high specificity and efficiency, typically operating under mild reaction conditions with lower energy input, resulting in both economic and environmental benefits [17]. Proteases and keratinases can be effectively produced by bacteria under optimized fermentation conditions, the determination of which is key to maximizing yield, process efficiency, and commercial profitability [17].

The keratinolytic potential of microorganisms is particularly valuable in the biodegradation of keratin-rich substrates such as feathers during composting. Several studies documented the effective composting of feather keratin, demonstrating its feasibility and agronomic value [2,9,18,19,20]. Keratin waste constitutes a valuable source of macro- and micronutrients, especially nitrogen, phosphorus, potassium, magnesium, and iron, making it an ideal substrate for producing eco-friendly, nutrient-rich organic fertilizers [14].

Microbial enzymatic activity is a key factor in keratin-rich compost, mediating the breakdown of organic matter. It can be monitored with indicators such as dehydrogenase and oxidoreductase activity, which are involved in carbon mineralization and the nitrogen cycle [21]. Such enzymatic markers reflect the dynamics and biological intensity of the composting process and can serve as indicators of compost maturity and stability [22].

There is a strong correlation between the degree of organic matter mineralization and the quality of the resulting compost, as the extent of mineralization determines its fertilizing potential. End products derived from keratin degradation also may be valuable across multiple industries, including agriculture (plant fertilizers), animal feed, pharmaceuticals, medicine, cosmetics, and cleaning products [23]. In recent years, increasing interest in biological waste valorization has supported the development of such biotechnologies as part of a circular economy strategy, aligned with the goals of the European Green Deal [24,25].

The study aimed to assess the impact of keratinolytic *Bacillus subtilis* inoculation on feather degradation and compost quality in solid-state cultures and composting trials at laboratory and semi-technical scales. It also aimed to characterize the strain’s keratinolytic enzymes and evaluate the fertilizing potential of the resulting compost.

## 2. Materials and Methods

### 2.1. The Keratinous Substrate

White broiler feathers were obtained from a local poultry-processing plant near Wrocław, Poland. Feathers were pretreated by washing with water and detergent, followed by degreasing in a 1:1 (*v*/*v*) mixture of methanol and chloroform. Subsequently, the material was rinsed with tap water and deionized water, and air-dried at room temperature and cut into 3–4 cm fragments. The prepared substrate was used in liquid cultures for evaluating the keratinolytic activity of the selected bacterial strain. Raw feathers, also cut to 3–4 cm fragments, were used in solid-state cultures and composts to better simulate the actual target process conditions. Lignite dust was obtained from the Sieniawa opencast mine (Poland) and sawdust sourced from deciduous trees.

### 2.2. Keratinolytic Bacteria

Keratinolytic bacterial strain *B. subtilis* P22, also referred to as *B. subtilis* PCM 2850, previously isolated from poultry feather waste, was applied in the study [26].

The inoculum for submerged cultures was prepared in nutrient broth medium (nutrient broth 8 g/L; glucose 10 g/L). Incubation was carried out in Erlenmeyer flasks at 30 °C for 12 h with shaking at 170 rpm. The resulting inoculum (1 × 10^9^ cfu/mL) constituted 2% *v*/*v* of the final experimental culture.

Liquid whey medium, based on food-grade components, was used to culture the *B. subtilis* P22 strain, which was subsequently applied as the 10% *v*/*v* inoculum for both the solid-state culture and the compost (1 × 10^9^ cfu/mL). The medium was composed of (g/L): demineralized whey powder (30.0), wheat germs (30.0), starch (30.0), and KH_2_PO_4_ (0.1), adjusted to pH 7.0 [27]. Whey was autoclaved at 117 °C at 0.8 atm for 15 min. Other medium components were sterilized separately at 121 °C at 1 atm for 30 min.

The inoculum for the solid-state culture was prepared in Erlenmeyer flasks and incubated at 30 °C for 12 h, with 170 rpm agitation. The inoculum for the compost process was cultivated in a 10 L stirred bioreactor (BioFlo^®^ 210, Eppendorf, Hamburg, Germany), in 6 L of medium. The process was conducted for 12 h at 30 °C, with 500 rpm stirring and 1 L/min aeration.

The bacterial strain *B. subtilis* P22 was stored on a nutrient agar slant at 4 °C.

### 2.3. Feather Degradation in Submerged Cultures

The keratinolytic bacterial strain *B. subtilis* P22 was cultivated in 250 mL conical flasks containing 100 mL medium, composed of (g/L): MgSO_4_·7H_2_O (1.0), KH_2_PO_4_ (0.1), FeSO_4_·7H_2_O (0.01), CaCl_2_ (0.1), yeast extract (0.5) and degreased chicken feathers (10.0). The cultures were incubated in a rotary shaker (170 rpm) for 7 days at 30°C. Finally, culture fluids were filtered out from insoluble residue with filter paper, centrifuged at 4000 rpm for 15 min at 4 °C, and the supernatant was stored at −24 °C for further analysis. The separated feather debris was dried at 105 °C and its dry mass was determined gravimetrically to calculate the residual feather content (%).

### 2.4. Feather Degradation in Solid State Cultures and Compost

Solid-state cultures and semi-technical compost batches were prepared from raw feathers (length of 3−4 cm), sawdust (particle size 3−4 cm), and lignite mixed at a ratio of 2:1:1 (*w*/*w*), respectively, to achieve an initial Corg/Nt ratio of 17. The total weight of the ingredients for the solid-state cultures was 2 kg, and for the semi-technical compost batches, 60 kg. The inoculum (1.0 × 10^9^ cfu/mL) constituted 10% of the total compost mass.

Uninoculated solid state culture and compost containing exclusively autochthonous microflora served as a control. Solid-state cultures were conducted in 1 L Roux bottles and maintained for 7 weeks. Semi-technical scale composting was performed over a period of 10 weeks using a closed, static reactor system with a volume of 200 dm^3^. The reactor was fabricated from polypropylene (PP) (a custom-built bioreactor, diameter 500/700 mm, height 890 mm, capacity 200 dm^3^) and included several key components: an airtight lid, an air supply sleeve, a carbon filter for the mitigation of odors, and an integrated port for monitoring temperature. The process utilized forced aeration, which was provided by an HL 275/50 compressor (Airpress Polska Sp. z o.o., Przeźmierowo, Poland) delivering a flow rate of 30 m^3^ per day. During composting, temperature, moisture content and pH were monitored. Temperature of the composted material was measured periodically (5 measurements in random locations, at a depth of 50 cm). pH was measured in 2 g samples, submerged in 20 mL of tap water, mixed for 30 min, and subjected to pH measurements [28]. The compost moisture content was adjusted to 60% every 7 days using tap water. At specified time points, samples were prepared for analysis as follows: for the solid-state cultures (laboratory scale), three independent samples were analyzed separately, whereas for the compost (semi-technical scale), three aliquots were pooled into a homogeneous mixture prior to the microbiological, enzymatic, physicochemical, and chemical analyses.

### 2.5. Determination of Proteolytic and Keratinolytic Activity

Soluble keratin was prepared according to Rodziewicz and Łaba [27]. Proteolytic [PU] and keratinolytic [KU] activity in culture fluids were determined with a modified method of Anson [27]. Casein solution (2%) (BDH) and soluble keratin (1 mg/mL) served, respectively, as substrates in the enzymatic reactions. One unit (1 U) of enzymatic activity was defined as the amount of enzyme required to release 1 μmol of tyrosine per 1 mL of culture fluid within 1 min, under specified experimental conditions (PU-protease activity, KU—keratinase activity; μmol·mL^−1^·min^−1^). All analyses were performed in triplicate.

### 2.6. Characterization of Proteases and Keratinases

The effect of pH of the reaction mixture on the protease and keratinase activity was tested at 30 °C in 0.05M Britton-Robinson universal buffer pH 6.0–10.5. The effect of temperature and pH on the protease activity was tested in the range of 35–65 °C, with 5 °C interval, at pH 7.0 in the same buffer. The sensitivity of proteases and keratinases to inhibitors and activators was tested in reference to: ethylenediaminetetraacetic acid disodium salt (EDTA, 2 mM), N-Ethyl maleimide (NEM, 5 mM), phenyl methyl sulfonic acid fluoride (PMSF, 2 mM), CaCl_2_ (10 mM), L-Cysteine (L-Cys, 5 mM) and L-Serine (L-Ser, 1 mM) [29]. After mixing with the enzymatic solution, protease and keratinase activity assays were performed under standard conditions. The percentage of inhibition or activation was calculated relative to the control:

% Inhibition = 100%−(I/C) 100%
% Activation = (A/C) 100%−100%, where
I—proteolytic activity in the presence of an inhibitor
A—proteolytic activity in the presence of an activator
C—proteolytic activity in the control sample

### 2.7. Measurement of Keratin Degradation Products

The following assays were used to measure the concentration of products of feather keratin degradation in submerged cultures: protein content with the method of Lowry, concentration of free amino groups of amino acids with TNBS method and concentration of free sulfhydryl groups with Ellman’s method [30]. All analyses were performed in triplicate.

### 2.8. Determination of Protease and Dehydrogenase Activity in Solid-State Cultures

The proteolytic activity was determined on 1% casein (BDH) with a modified method of Ladd and Butler [4,31] and expressed in μmol of tyrosine released within one minute per 1 g of compost d.m. (proteolytic unit—PU; µmol·g dm^−1^·min^−1^). To perform this assay, 10 mL of a 1% casein solution in 0.1 M Tris/HCl buffer with pH 8.1 were added to 2 g of compost. The reaction was conducted at a temperature of 50 °C for 20 min, and then stopped using a 12.5% solution of trichloroacetic acid (TCA). The samples were centrifuged and their absorbance was measured at the wavelength of λ = 280 nm against the control sample. Dehydrogenase activity was determined with a modified method of the Casida [4] method for 2,3,5-triphenyl tetrazolium chloride (TTC) and expressed in μmol of 1,3,5-triphenylformazan (TPF) formed within 20 h per 1 g of compost d.m. (dehydrogenase unit—DU; μmol TPF·g^−1^ dm·20 h^−1^). All analyses were performed in triplicate.

### 2.9. Chemical Analysis of Compost

Mineral and fertilizer analyses of selected compost samples and their individual components were carried out at the Institute of Soil Science and Environmental Protection, University of Life Sciences in Wrocław. The study included determinations of individual fractions of carbon, nitrogen, and sulfur, with organic carbon (Corg) measured by the oxidimetric method using K_2_Cr_2_O_7_. The carbon content of specific groups of organic compounds was assessed through fractional analysis according to Stevenson [32]:

Cw—water-soluble carbon in water with a sample to water mass ratio of 1:20,
Ck—carbon soluble in sulfuric acid (VI) (proteins, hemicellulose, fulvic acids) extracted with 5% sulfuric acid at 80 °C,
Cbit—compounds from the bitumen group extracted with a mixture of ethyl alcohol:benzene (1:2).

Total nitrogen (Nt) was determined by Kiejdahl method (Kiejdahl apparatus, BÜCHI Labortechnik AG, Flawil, Switzerland). Ammonium and nitrate nitrogen (N-NH_4_ and N-NO_3_) concentration were measured spectrophotometrically in water extracts 1:100. Total sulfur (St) was determined in the CS-MAT 5500 instrument (Strohlein GmbH & Co., Kaarst, Germany, currently Bruker AXS Inc., Madison, WI, USA). The total contents of selected macroelements (P, K, Mg, Na, Ca) and microelements (Ni, Fe, Cr, Pb, Zn, Mn, Cu, Cd) were determined by the ICP method (ICP-AES Thermo Scientific iCAP 7400, Waltham, MA, USA) after mineralization (Microwave Digestion System—Start D, Milestone S.r.l., Sorisole, Italy) with a mixture of concentrated hydrochloric and nitric acid in a volume ratio of 3:1. The total Hg content was measured on an AMA analyzer (MA-2, Nippon Instruments Corporation, Kyoto, Japan). Results of analyses were interpreted based on averaged values of 2 replications.

### 2.10. Statistical Analysis

Statistical analyses were conducted to compare experimental groups with corresponding controls. Pairwise comparisons were performed using Student’s *t*-test, or Welch’s *t*-test when the assumption of equal variances was not met. Statistical significance was assessed at three thresholds (*p* < 0.05, *p* < 0.01, and *p* < 0.001), which are indicated in the figures.

## 3. Results and Discussion

### 3.1. Degradation of Feather Keratin in Submerged Culture

**Figure 1:**
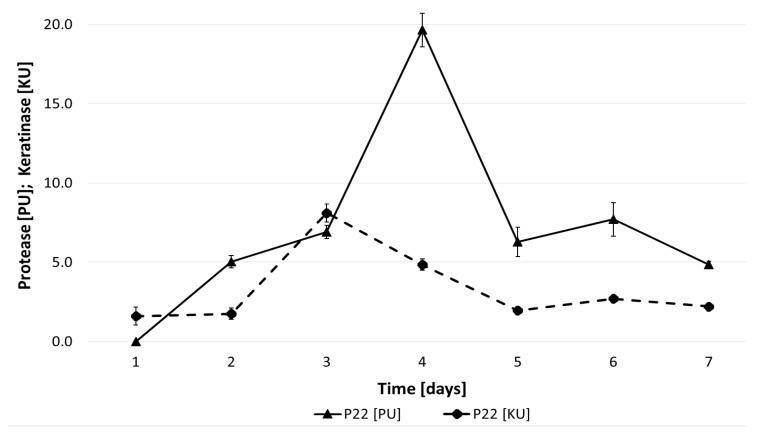
Proteolytic and keratinolytic activity of *B. subtilis* P22 during cultivation in feather-containing medium. Data values are presented as mean ± SD.

*Bacillus subtilis* P22 exhibited growth in the medium with chicken feathers as the sole carbon source, and its production of proteolytic and keratinolytic enzymes was initiated at the onset of the stationary phase (days 3–4), consistent with observations reported for *B. subtilis*, *B. pumilus*, and *B. cereus* by Kim et al. [33]. Maximum proteolytic activity (20 U/mL) was detected on day 4, whereas peak keratinolytic activity (8 U/mL) occurred on day 3 (Figure 1). However, keratin decomposition is not limited to enzymatic hydrolysis. Korniłłowicz–Kowalska [34] demonstrated that, in addition to proteolytic and keratinolytic activity, several parameters can serve as indicators of microbial keratinolytic potential, including culture medium alkalization, the release of soluble proteins and amino groups, the formation of oxidized sulfur compounds, and the reduction in feather mass [14]. Besides keratinases and proteases, which degrade recalcitrant proteins, oxidoreductive enzymes such as disulfide reductases, as well as reducing agents including sulfide, sulfite (SO_3_^2−^), glutathione, and cysteine, may also be involved. Cleavage of disulfide bridges leads to the accumulation of sulfur in the form of –SH groups, which are subsequently oxidized to sulfite (SO_3_^2−^), sulfate (SO_4_^2−^), thiosulfate (S_2_O_3_^2−^), and organic thiol compounds. In their studies on keratinolysis, Rodziewicz and Łaba [27] concluded that, in addition to proteolytic enzymes, other extracellular metabolites released during feather degradation in submerged cultures contributed to the process. Among these, disulfide reductases and other reducing compounds facilitating the cleavage of cystine disulfide bonds played a central role. Their results showed a sequential release of metabolites, including thiol groups (1.40 mM), sulfite (0.8 mM), and sulfate (2.6 mM), supporting the conclusion that disulfide bond cleavage is a fundamental step that proceeds in parallel with proteolytic degradation.

**Figure 2:**
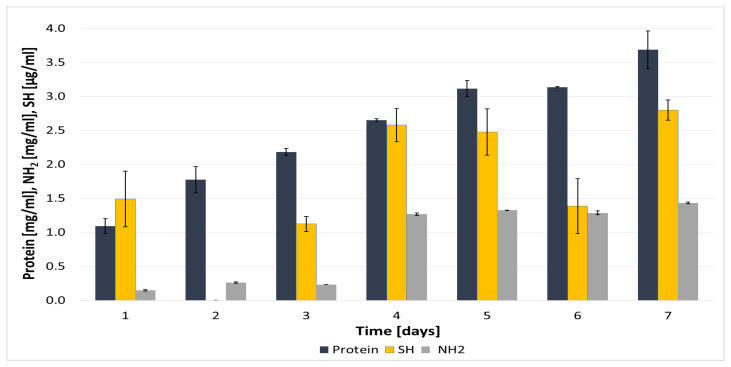
Accumulation of soluble proteins, reduced thiols, and free amino groups during cultivation of *B. subtilis* P22 in feather-containing medium. Data values are presented as mean ± SD.

Keratinolysis in the culture of *B. subtilis* P22 was accompanied by a gradual accumulation of degradation products, including free amino groups (1.4 mg/mL), thiol groups derived from cysteine through the reduction in cystine disulfide bridges (2.8 mg/mL), and soluble protein (3.6 mg/mL) (Figure 2). A similar sequential release of proteins (2.98 mg/mL), sulfhydryl groups (2.14 × 10^−4^ M), and free amino acids (3.24 mg/mL) was reported by Gurav et al. [35] during feather keratinolysis. The progressive accumulation of amino acids and peptides may act as a regulatory signal, potentially inhibiting further hydrolysis and keratinase synthesis through feedback mechanisms.

The loss of feather dry mass after cultivation was the indirect measure of strain keratinolytic capabilities. *B. subtilis* P22 achieved 78% liquefaction of feather dry mass after 7 days at 30 °C. Keratinolytic activity has been extensively described in bacteria, particularly among *Bacillus* spp., including industrially relevant strains such as *B. subtilis*, *B. cereus*, *B. stearothermophilus*, *B. pumilus*, and *B. licheniformis* [36,37,38,39,40]. A comparable screening approach has been reported, e.g., by Nagarajan et al. [41], who investigated keratinolytic isolates from soil at a poultry feather dumping site. In contrast, the *Bacillus* sp. strain described by Riaz et al. [16] achieved almost complete degradation (98%) after 7 days at 45 °C; however, such thermophilic conditions are associated with higher process costs compared to cultivation at 30 °C. Łaba et al. [42] identified *Kocuria rhizophila* PCM 2931, capable of degrading up to 52% of chicken feathers within 4 days under optimized conditions.

**Figure 3:**
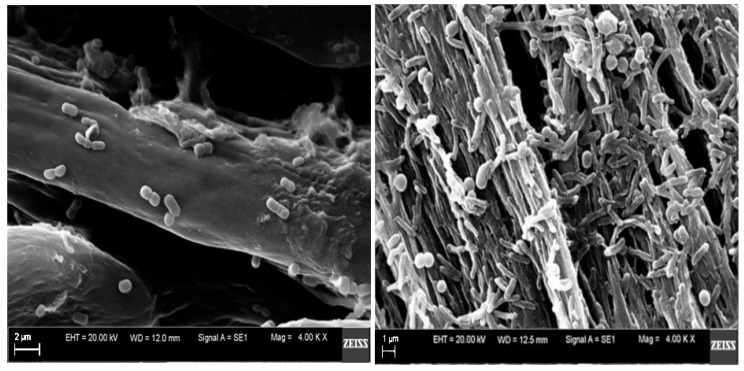
SEM images focusing on barb structure of feathers on day 0 (**left**—scale bar 2 µm) and day 7 (**right**—scale bar 1 µm) of *B. subtilis* P22 submerged culture (magnification × 4.00 K).

Scanning electron microscopy (SEM) provided detailed visualization of the feather barbs surface during degradation by *B. subtilis* P22 (Figure 3). Numerous bacterial cells were observed adhering to the feather ray surface, indicating effective colonization and the onset of biodegradation. The direct contact between bacterial cells and the keratin matrix created favorable conditions for the enzymatic action at the cell–substrate interface. Progressive erosion of the barb surface was evident, accompanied by the formation of numerous cracks and partial exposure of keratin fibers, consistent with enzymatic degradation of the feather structure. These morphological alterations corroborate the keratinolytic potential of the strain and align with the results of biochemical analyses.

### 3.2. Characterization of Proteases and Keratinases of B. subtilis P22

During composting of keratinous waste, keratinases—specialized proteolytic enzymes, predominantly serine proteases—play a central role. Understanding their properties and optimal reaction conditions is critical for efficient hydrolysis and process control. The genome of *B. subtilis* harbors multiple peptidase genes, including 14 intracellular, 22 membrane-bound, and 8 extracellular proteases. The latter subset is typically secreted in response to induction by proteinaceous substrates present in the growth environment [43]. As demonstrated by Mazotto et al. [44], eight activity bands with various molecular weight were present in the casein zymogram of *B. subtilis* AMR, however strongly induced by keratin and yeast extract. Among them, 3 peptidases demonstrated strict activity towards feather keratin.

**Figure 4:**
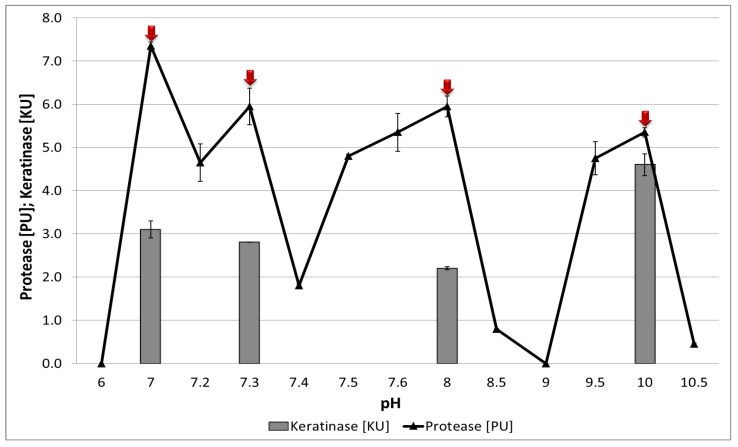
The influence of environmental pH on the activity of proteolytic and keratinolytic enzymes of the *B. subtilis* P22 strain. Data values are presented as mean ±SD. Red arrows indicate the pH values at which the highest proteolytic activity was obtained.

In our study, enzyme characterization was conducted using crude culture fluid of *B. subtilis* P22. The effect of pH on protease and keratinase activity was examined at 30 °C. Peaks of proteolytic activity were observed at pH 7.0, 7.3, 8.0, and 10.0, with maximum activity at pH 10.0, suggesting dominance of alkaline serine proteases (subtilisins) (Figure 4). Keratinases are often characterized by broad pH tolerance, as exemplified by the *B. megaterium* keratinase described by Park et al. [45], which retained activity from pH 7 to 11, with a maximum at pH 7–8. Similar findings were reported by Moussa et al. [46] for alkaline keratinases from *Bacillus* sp. D4, which exhibited the highest activity at pH 8.0 (54.3 U/mL). Different observations were reported by Al-Bedak et al. [13] for enzymes released by the keratinolytic fungal strain *Didymella keratinophila* AUMC 15399, which showed the highest keratinase activity at pH 7 (5.8 U/g).

**Figure 5:**
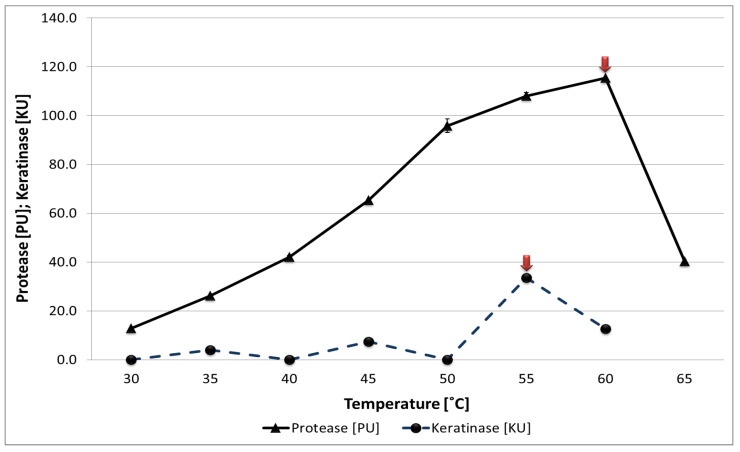
The influence of temperature on the activity of proteolytic and keratinolytic enzymes of the *B. subtilis* P22 strain. Data values are presented as mean ± SD. Red arrows indicate the temperature values at which the highest proteolytic and keratinolytic activity were obtained.

Reaction temperature proved to be the most critical parameter affecting the activity of both proteases and keratinases, as its optimization led to a several-fold increase in the activity of both enzyme groups (Figure 5). Proteases secreted by the *B. subtilis* P22 strain exhibited the highest activity of 115 U/mL at 60 °C, while keratinases reached a maximum activity of 33 U/mL at 55 °C. The most intensive keratin decomposition under composting conditions is therefore expected to occur during the thermophilic phase, driven by extracellular keratinases that remain active after being synthesized prior to the temperature peak, despite the mesophilic nature of the producing strain. This observation contrasts with reports by Gupta and Ramnani [47], who suggested that efficient bacterial keratinolytic activity is typically observed at mesophilic temperatures (25–37 °C). Mousa et al. [46] described keratinases with optimal activity at 37 °C (55.0 U/mL), with activity gradually declining above 40 °C, and little or no bacterial growth beyond 55 °C.

The catalytic properties of proteolytic enzymes can be characterized by assessing their response to specific activators and inhibitors. In our study, proteases showing maximal activity at neutral pH values (7.0–7.3) were strongly inhibited (>70%) by EDTA, NEM, and PMSF, but activated (56–278%) by calcium ions and cysteine (Table S1). These findings indicate a polycatalytic enzyme system (P) comprising metalloproteases (M), thiol proteases (T), and serine proteases (S). Comparable proteolytic conditions (pH 7.0, 60 °C) were reported by Suh and Lee [48], who characterized a purified keratinase from *B. subtilis* KS-1. That enzyme was inhibited by PMSF, confirming its classification as a serine protease. Also, Mazotto et al. [49] demonstrated maximum activity of keratinase from *B. subtilis* at pH 10 and 50 °C, identified as a serine peptidase due to PMSF inhibition. In the same study, the maximum activity of gelatinolytic proteases was shifted towards pH 9.0 and 60 °C. In line with these findings, proteases in the present study displayed maximal activity under alkaline conditions (pH 8.0–10.0), with the highest activity recorded at 60 °C. These enzymes exhibited polycatalytic (P) behavior, likely consisting of a mixture of T, S, and M proteases, with thiol proteases predominating, as serine proteases were further activated by serine (49%) at pH 10.0.

Numerous studies have demonstrated that keratinases produced by the genus *Bacillus* belong primarily to serine proteases [14,49]. However, other enzymes such as disulfide reductases, trypsin, and peptidases, which act synergistically, are also believed to contribute to keratin hydrolysis [50].

### 3.3. Degradation of Feather Keratin in Solid-State Culture

**Figure 6:**
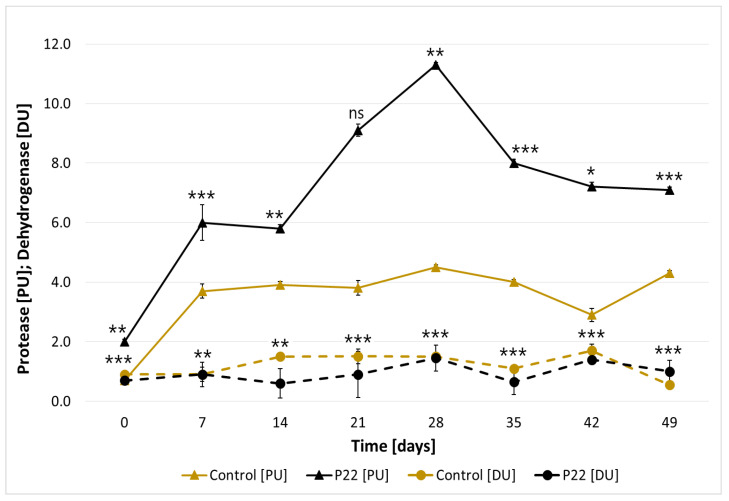
Protease (PU) and dehydrogenase (DU) activity in solid-state culture of *B. subtilis* P22 compared to the uninoculated control over 49 days of incubation. Data values are presented as mean ± SD. Statistical differences were assessed using pairwise comparisons (Student’s *t*-test) between the P22 culture and the control for each time point, performed separately for PU and DU. Significant differences are indicated as *p* < 0.05 (*), *p* < 0.01 (**), *p* < 0.001 (***), non-significant (ns).

**Figure 7:**
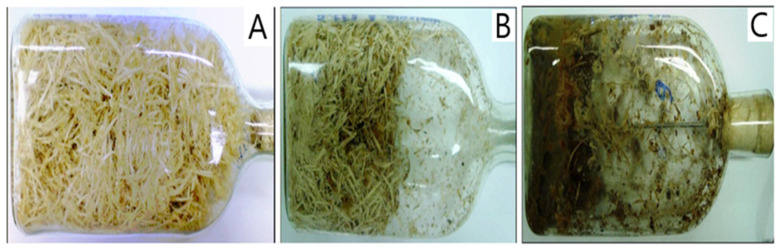
Macroscopic evaluation of bristle biodegradation in solid-state culture: initial mixture (**A**), uninoculated culture—control (**B**), inoculated culture (**C**).

The activity of proteases in inoculated composts was significantly higher compared to composts containing only indigenous microflora (Figure 6). The peak activity of these enzymes occurred in the 4th week of the process, reaching 11.3 [PU]. A different trend was observed for redox enzymes, represented by dehydrogenases. In this case, the highest activity (1.7 DU) was recorded in the control, uninoculated compost (Figure 6). Nevertheless, the final dry mass loss in the inoculated sample (44.7%) was almost twice that of the uninoculated culture (23.9%). This indicates a higher efficiency of organic matter degradation in the inoculated compost, further supported by the visual comparison of the material before and after composting (Figure 7).

Bohacz and Korniłłowicz-Kowalska [51] studied composting of feathers combined with lignocellulosic waste (bark and straw) in four different composition variants. The process, conducted for 7 months with only autochthonous microflora, included feathers at a 10% proportion of the compost mass. After 10 weeks, the reduction in organic dry matter reached 60%. It should be emphasized that in the composts analyzed in the present study, the keratin substrate content was five times higher, yet after only seven weeks the dry mass loss reached 44.7%. In this context, the obtained results appear promising.

Extracellular enzymes secreted by microorganisms are essential for the decomposition of organic matter during composting [52]. They include enzymes involved in carbon cycling (cellulases, xylanases, chitinases, lipases), nitrogen cycling (aminohydrolases, ureases), and phosphorus cycling (phosphatases), as well as redox enzymes such as dehydrogenases, which reflect overall microbial metabolic activity [53]. Mondini et al. [54] demonstrated a clear relationship between enzymatic activity and both the quantity and quality of organic matter, suggesting that enzyme activity can serve as a criterion for compost maturity, defined as the extent of biodegradable substrate decomposition. In soils, extracellular enzymes show limited stability due to denaturation, inactivation, and proteolysis. However, humic substances form buffer systems that can stabilize enzymes and mitigate their degradation. A comparable effect can be expected during composting, where the gradual increase in humus content may enhance enzyme persistence under unfavorable environmental conditions.

### 3.4. Composting of Feathers on Semi-Technical Scale

**Figure 8:**
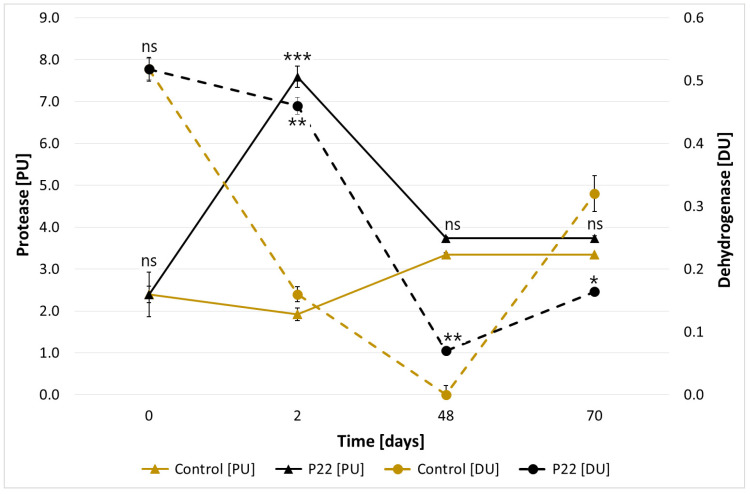
Protease (PU) and dehydrogenase (DU) activity in compost inoculated with *B. subtilis* P22 compared to uninoculated control, over 70 days of incubation. Data values are presented as mean ± SD. Statistical differences were assessed using pairwise comparisons (Student’s t-test) between the P22 culture and the control for each time point, performed separately for PU and DU. Significant differences are indicated as *p* < 0.05 (*), *p* < 0.01 (**), *p* < 0.001 (***), non-significant (ns).

Solid-state cultivation of the tested strain on keratin-lignocellulose biomass justified scaling up of the experiment. At the semi-technical scale conducted in 200 dm^3^ reactors, both protease and dehydrogenase activities decreased in inoculated and control composts compared to solid-state culture values (Figure 8). Sutripta et al. [55] reported that inoculation of vegetable waste compost with thermophilic *Geobacillus* sp. increased dehydrogenase activity and lowered the C:N ratio, confirming inoculum effectiveness. Composting remains an efficient method for recycling biogenic elements from biodegradable organic matter. During the 2 months, clear differences were observed between inoculated and control composts (Table 1). Inoculation with *B. subtilis* P22 enhanced decomposition of organic matter, as indicated by reduced organic carbon (Corg 229.9 g/kg), bituminous fraction (Cbit 9.0 g/kg), and sulfuric acid-soluble carbon (Ck 2.7 g/kg), whereas water-soluble carbon (Cw 2.6 g/kg) was higher in the control. These results suggest that *B. subtilis* P22 accelerates carbon transformation and promotes more advanced compost maturation compared to the uninoculated control.

A distinctive effect of *B. subtilis* P22 inoculation was the modification of nitrogen dynamics (Table 1). The inoculated compost showed higher total nitrogen (Nt, 2.20 g/kg) and nitrate (N-NO_3_^−^, 2.45 mg/g), accompanied by lower ammonium nitrogen (N-NH_4_^+^, 0.88 mg/kg). This pattern of declining N-NH_4_^+^ and accumulating N-NO_3_^−^ is a clear indicator of proper compost maturation and reflects intensified nitrification processes. Such a trend is desirable, as it demonstrates efficient mineralization of organic matter and stabilization of nitrogen into a readily plant-available form, while simultaneously reducing the risk of ammonia volatilization. As a consequence, the fertilizer value of the compost is enhanced. Similar shifts in N-NO_3_^−^ and N-NH_4_^+^ have been reported as reliable markers of compost maturity [57].

The increase in the ammonia oxidation process is related to the increasing temperature of composts and the increasing pH of the compost mass [58]. Jordar et al. [20] described in their work the composting of feathers alone in the soil for a period of 3 months. However, it should be noted that in addition to feathers, the compost also contained sawdust, which constituted 25% of the compost and contained a negligible nitrogen content. Therefore, the content of this element in the feathers subjected to microbiological treatment was significantly higher. The guidelines in force in EU countries regulating the limits of the content of individual macro- and microelements can be found in Regulation (EU) Regulation (EU) 2019/1009 of the European Parliament and of the Council, 5 June 2019, laying down rules on making the market of EU fertilising products available and amending Regulations (EC) No 1069/2009 and (EC) No 1107/2009 and repealing Regulation (EC) No 2003/2003 [56].

The contents of two important components determining fertilizer efficiency, K_2_O and P_2_O_5_, were significantly higher in the inoculated compost and met regulatory standards (Table 1). Phosphorus content reached 2.06 g/kg, while potassium content was nearly doubled (2.27 g/kg) compared with the control, confirming the positive effect of inoculation. Keratin proteins are rich in sulfur-containing amino acids, which, metabolized by microbial cells, are released as inorganic sulfur compounds. Previous studies identified sulfur content in feather–lignocellulosic composts as a useful maturity indicator [51]. In the present study, the sulfur content of inoculated compost was 20% higher than in the uninoculated control, confirming enhanced keratin degradation and sulfur transformation.

Compost maturity is commonly assessed using established indices such as the C/N and N-NH_4_^+^/N-NO_3_^−^ ratios. Numerous studies propose C/N < 12 as a maturity threshold [59,60]. A higher C/N ratio (C/N > 25:1) leads to the immobilization of mineral nitrogen, for example, through its incorporation from the soil into microbial biomass [61]. According to this criterion, only the inoculated compost reached maturity, while the control slightly exceeded the limit (Table 2). A similar pattern was observed for the Cw/Corg index. Regarding nitrogen transformations, both composts achieved maturity, but the inoculated compost displayed a lower N-NH_4_^+^/N-NO_3_^−^ ratio (0.4), reflecting a greater prevalence of nitrate over ammonium, the form more desirable for fertilization purposes.

Mineral composition also supports the fertilizer potential of the inoculated compost [62]. These include calcium (0.06 ppm), phosphorus (0.05 ppm), potassium (0.04 ppm), sodium (0.04 ppm), magnesium (0.03 ppm), manganese (0.01 ppm), iron (0.01 ppm), and zinc (0.01 ppm). In our study, inoculated composts showed substantially higher concentrations: calcium (0.67%), magnesium (0.83%), and iron (0.98%) (Table 3). Comparable levels of Ca (0.8%) and Mg (0.7%) were reported by Jordar et al. [20] in feather-only compost. In terms of the heavy metals content (Pb, Cd, Hg), both inoculated and control composts met regulatory requirements.

In the compost samples tested, the control sample exhibited a total chromium concentration of 4.6 mg/kg, while the inoculated sample contained 5.1 mg/kg (Table 3). Total chromium analysis, typically performed using techniques such as Inductively Coupled Plasma (ICP), provides a cumulative measurement covering both Cr(III) and Cr(VI), as this method does not differentiate between oxidation states. However, Cr(III) is the predominant species in most environmental matrices [63,64]. In natural soils, hexavalent chromium (Cr(VI)) generally accounts for only a small fraction of total chromium due to its high reactivity and tendency to be reduced to Cr(III) in the presence of organic matter and common soil reductants [65]. Although Cr(VI) is recognized for its higher toxicity, it can readily undergo reduction in environmental conditions. Therefore, it is unlikely that the total chromium concentrations determined via ICP exceed the regulatory thresholds listed in the table.

Temperature measurements of the compost mass serve as a reliable indicator of microbial metabolic activity under varying conditions (Figure S1). In both the inoculated and control composts, the temperature profiles were comparable. The thermophilic phase, characterized by temperatures exceeding 45 °C, commenced on the third day and lasted for 10 consecutive days. The maximum temperature observed in the inoculated compost reached 57 °C. Enzymatic assays revealed that the optimal activity temperatures for proteases and keratinases secreted by *Bacillus subtilis* P22 were 60 °C and 55 °C, respectively. Therefore, the degradation of keratinous substrates is expected to be most effective during the thermophilic phase of composting, when temperatures remain within the optimal range for these enzymes. Additionally, a rise in pH was observed during this phase (Figure S1), with both compost variants reaching similar alkaline values (pH 8.0–8.5). This alkalinization likely reflects the metabolic activity of keratinolytic microorganisms in protein-rich environment, including both the indigenous microbial community and the introduced *B. subtilis* P22 strain.

Scalability and potential limitations are critical considerations when translating small-scale composting studies to industrial applications. Experimental simulations at reduced scales may lack reliability, as the organic mass is often insufficient to reproduce the thermal inertia of full-scale systems [66]. Lashermes et al. [67] investigated sewage and plant waste composting in laboratory reactors (<10 L), aiming to design an innovative small-scale device and evaluate process efficiency and reproducibility. The study showed that organic matter losses, compost stabilization, and cellulose and hemicellulose transformations were comparable to those in larger systems, except for lignin degradation. Thermophilic conditions developed spontaneously in all reactors, indicating that composting performance observed at the semi-technical scale can likely be achieved at the industrial level.

A major limitation in applying compost inocula is the cost of their preparation, transport, and storage. Using low-cost substrates, including agri-food by-products, in inoculum media could significantly reduce production costs. Due to the complexity of inoculum production, preservation methods such as freeze-drying or spray-drying should be considered to ensure strain viability, maintain process continuity, and simplify storage and distribution.

As an alternative, backslopping, i.e., the use of a portion of mature compost to inoculate fresh biomass, can reduce overall process costs by partially eliminating the need for repeated large-scale inoculum preparation. However, this approach carries the risk of losing the target strain or prevalence of undesirable microorganisms, highlighting a trade-off between cost reduction and process reliability that must be carefully assessed. In this context, the use of *B. subtilis* strain P22, a spore-forming bacterium, is advantageous, as its spores remain viable throughout the thermophilic phase of composting, ensuring its persistence.

Variability in feather waste composition may also present challenges, warranting further large-scale studies. Given the dynamic nature of composting, developing effective monitoring methods and enabling supplementary inoculation during the process could help prevent disruptions in biomass decomposition.

## 4. Conclusions

This study demonstrated that *Bacillus subtilis* P22 effectively degraded poultry feathers through the secretion of polycatalytic proteolytic enzymes, predominantly alkaline serine proteases. Compost inoculated with *B. subtilis* P22 exhibited more intense transformations of mineral compounds and a higher degree of organic matter degradation than the uninoculated control, as confirmed by key maturity indicators (C/N, NH_4_^+^/NO_3_^−^, and Cw/Corg ratios). The inoculated compost also exhibited improved mineral composition, particularly in terms of nitrogen, potassium, phosphorus, and sulfur content. The enriched mineral composition of the mature biomass suggests its potential utility as an organic soil improver in agricultural applications; however, this potential should be further validated through plant growth and crop performance experiments under field conditions.

## Figures and Tables

**Table 1:** Mineral composition of semi-technical compost.

Compost	Corg	Cbit	Cw	Ck	St	Nt	N-NH_4_	N-NO_3_	K_2_O	P_2_O_5_
	g/kg									
Control	237.0	10.2	2.6	2.0	5.2	19.4	1.15	1.50	1.13	1.83
Inoclulated	229.9	9.0	2.1	2.7	6.1	22.6	0.88	2.45	2.27	2.06
Desired trend *	>7.5% *	↓	↑	↑	↑	↑	↓	↑	↑	↑

* According to Regulation (EU) 2019/1009 of the European Parliament and of the Council, 5 June 2019, laying down the rules on making the market of EU fertilising products available and amending Regulations (EC) No 1069/2009 and (EC) No 1107/2009 and repealing Regulation (EC) No 2003/2003 (for organic soil improver) [56]. The arrows represent the desired direction of changes of a given parameter in the composting process.

**Table 2:** Compost maturity indicators in semi-technical compost.

Compost	C/N	N-NH_4_/N-NO_3_	Cw/Corg
	-	-	[%]
Control	12.2	0.8	1.1
Inoculated	10.2	0.4	0.9
Desired trend	↓ <12	↓ <1	↓ <1%

The arrows represent the desired direction of changes of a given parameter in the composting process.

**Table 3:** Macro and microelements content in semi-technical compost.

Compost	Ca	Mg	Fe	Mn	Na	Ni	Cr	Cd	Hg	Pb	Zn	Cu
	g/kg					mg/kg						
Control	6.0	0.7	8.7	0.1	0.8	5.4	4.6	nd *	nd *	16.1	62.3	7.4
Inoculated	6.7	0.8	9.8	0.1	1.2	6.8	5.1	nd *	nd *	19.6	65.8	8.9
Desired trend ***	↑	↑	↑	↑	↑	<50	<2 **	<2	<1	<120	<800	<300

* nd—not detected. ** hexavalent chromium (Cr VI). *** According to: Regulation (EU) 2019/1009 of the European Parliament and of the Council, 5 June 2019, laying down rules on making the market of EU fertilising products available and amending Regulations (EC) No 1069/2009 and (EC) No 1107/2009 and repealing Regulation (EC) No 2003/2003 (for organic soil improver). The arrows represent the desired direction of changes of a given parameter in the composting process.

## Data Availability

The original contributions presented in this study are included in the article. Further inquiries can be directed to the corresponding author.

## Supplementary Materials

The following supporting information can be downloaded at https://www.mdpi.com/article/10.3390/ma18204667/s1. Figure S1: Temperatures and pH of inoculated and control compost throughout the composting process; Table S1: Influence of inhibitors (% inhibition) and activators (% activation) on the enzymatic activity of proteases and reactions performed at pH values corresponding to previously determined activity peaks at a temperature of 60 °C.

## Abbreviations

The following abbreviations are used in this manuscript:

PMSF	Phenyl methyl sulfonic acid fluoride
NEM	N-Ethyl maleimide
EDTA	Ethylene diamine tetraacetic acid disodium salt
