# Supplementary material for: Composting Poultry Feathers with Keratinolytic Bacillus subtilis: Effects on Degradation Efficiency and Compost Maturity
Source: Materials (Basel). 2025 Oct 11;18(20):4667. doi: 10.3390/ma18204667 (PMC12565654; doi:10.3390/ma18204667)
Supplement: Supplementary file 1 [file materials-18-04667-s001.zip › materials-3899673-supplementary.pdf]

*Supplementary Materials*

# Composting Poultry Feathers with Keratinolytic *Bacillus subtilis*: Effects on Degradation Efficiency and Compost Maturity

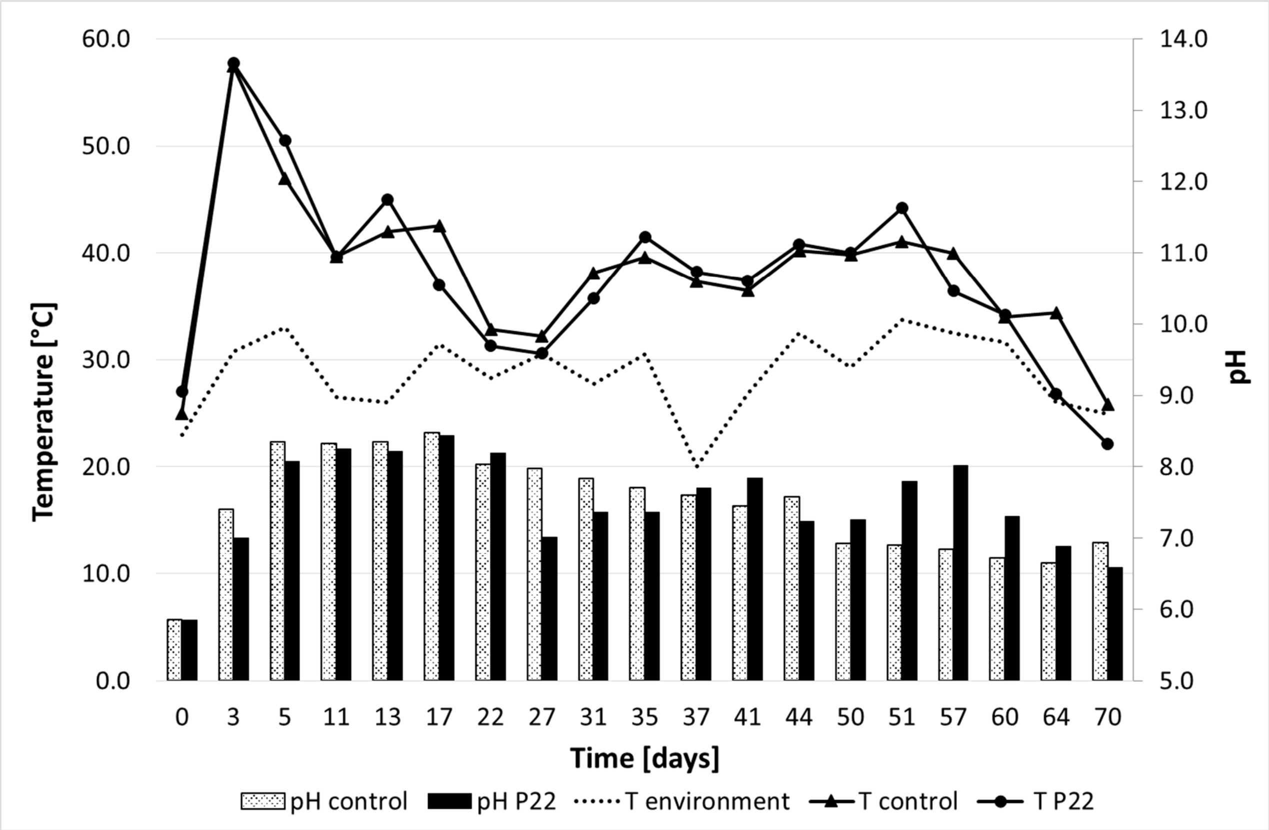


**Figure S1.** Temperatures and pH of inoculated and control compost throughout the composting process.

**Table S1.** Influence of inhibitors (% inhibition) and activators (% activation) on the enzymatic activity of proteases; reactions performed at pH values corresponding to previously determined activity peaks, at temperature 60°C.

| pH | INHIBITION | | | ACTIVATION | | |
|---|---|---|---|---|---|---|
| | EDTA [2mM] | NEM [5mM] | PMSF [2mM] | $CaCl_2$ [10mM] | L-Cys [5mM] | L-Ser [1mM] |
| | Inhibition/ Activation [%] | | | | | |
| **7** | 93±1.1 | 100±0.6 | 100±6.0 | 100±8.1 | 56±2.5 | - |
| **7.3** | 78±1.2 | 100±0.7 | 100±0.6 | 224±0.0 | 278±6.9 | - |
| **8** | 36±2.9 | 97±0.1 | 75±3.4 | 81±0.4 | 126±2.9 | 0±0.0 |
| **10** | 49±5.5 | 100±1.9 | 46±6.2 | 74±17.8 | 26±3.9 | 49±7.5 |
